# Selected Properties of Self-Compacting Concrete with Recycled PET Aggregate

**DOI:** 10.3390/ma15072566

**Published:** 2022-03-31

**Authors:** Justyna Jaskowska-Lemańska, Milena Kucharska, Jakub Matuszak, Paweł Nowak, Wojciech Łukaszczyk

**Affiliations:** Faculty of Civil Engineering and Resource Management, AGH University of Science and Technology, 30-059 Cracow, Poland; lemanska@agh.edu.pl (J.J.-L.); jakub.matuszak70@gmail.com (J.M.); pawelsams.pn@gmail.com (P.N.); wojtek.tady@gmail.com (W.Ł.)

**Keywords:** self-compacting concrete, PET, recycled aggregate, eco-efficient concrete, fresh properties of SCC mechanical properties, non-destructive tests (NDT)

## Abstract

In this paper, the issue of self-compacting concrete (SCC) with the addition of polyethylene terephthalate (PET) recycled aggregate is addressed. The PET utilized was a waste fraction in the PET-bottle-recycling process. The implementation of waste in concrete mixes has a positive impact on their environmental and social profile; however, technical requirements are not necessarily met. In this investigation, PET was used as a substitute for fine aggregate in quantities ranging from 0 to 20% in increments of 5%. Both the flow properties of SCC mixes and the hardened SCC properties (compressive strength, splitting tensile strength, modulus of elasticity, and Poisson ratio) were investigated. Additionally, non-destructive tests (ultrasound and sclerometric) were performed to determine the correlation curves. The research revealed that both the flow properties and the parameters of the hardened concrete deteriorated with the PET content. Concrete with 20% PET replacement did not meet the self-compacting requirements and its compressive strength decreased by almost 50%. However, it was noted that replacing fine aggregate with PET aggregate in the amount of 5% did not significantly alter the concrete parameters and could be an attractive alternative to traditional concretes. Based on non-destructive testing, correlation curves were constructed that could be applicable to the future quality assessment of self-compacting concretes with recycled PET aggregate.

## 1. Introduction

The rapid consumption of multiple natural resources and the production of substantial amounts of waste by the construction industry are widely recognized to contribute to a serious environmental problem. With the development of both science and technology, including building materials, this crisis should be continuously alleviated. One of the prevailing approaches is the implementation of intelligent and energy-efficient solutions in the construction sector, such as the idea of a circular economy or new generation materials with improved durability parameters.

Today, concrete is the most widely used construction material due to its versatility, good general availability, favorable mechanical properties, and relatively low price. Its production increases annually (approximately 32 billion tons worldwide [1]) and is unfortunately associated with harmful emissions of carbon dioxide (CO_2_) and particulate matter (PM_10_) to the environment, as well as the depletion of natural resources. Following the sustainable development strategy, new technologies have been introduced to the construction industry for several decades, for example, eco-efficient concrete. These measures aim, among other things, to minimize the environmental impact during production, increase the durability and resistance to environmental hazards, extend structural reliability, and reduce the life cycle costs of structures.

In the world literature, suggested strategies for the development of eco-efficient concrete involve, among others, the replacement of Portland cement’s (which is responsible for approximately 8% of total CO_2_ emissions [1]) mineral additives and natural aggregates with postproduction waste (e.g., recycled aggregate) or a general reduction in the contribution of the binder to the composition [2,3,4]. Thus, the idea of using eco-efficient concrete can help reduce harmful emissions of CO_2_ and PM_10_, limit the consumption of natural mineral resources, and reduce post-industrial waste, which can be given a “second chance of life,” in line with the concept of a circular economy. In their final life cycle stage, these materials, such as traditional concrete, can be recycled by preparing crushed concrete to replace natural aggregate, as a highway substrate, or as a clean fill around buildings. Compared to other materials, concrete is relatively environmentally friendly; therefore, the eco-improvement of the performance characteristics of concrete must be multiphased and customized to the location of its implementation. The analysis of applied solutions taking into account the whole life cycle of the building object is considered crucial for concrete construction [5,6,7]. Therefore, the influence of design strategies on environmental and economic efficiency [8], as well as solutions favorable to the creation of an environmental profile, are being investigated.

Another global concern is the depletion of natural raw materials, including sand, which is used extensively in the construction, road, and glass industries. Although Earth’s sand resources are relatively abundant, not all sand is suitable for concrete mixes. Thus, in concrete technology, the concept of substituting sand with recycled aggregates, including glass or plastic products, emerged [9].

**Figure 1:**
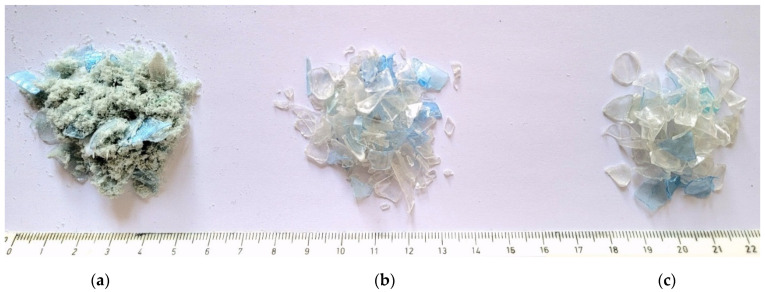
PET bottle recycling products: (**a**) PET fines waste/powder—manufacturing waste, (**b**) washed PET fines of fraction 0.5–4.0 mm—non-quality product, and (**c**) washed PET Fines of fraction 4.0–12.0 mm—quality product.

Due to their superior properties (lightweight, relatively high strength, flexibility in transforming into different shapes, and high resistance to bacteria), plastics remain in use in many industries around the world. In the last 50 years, plastic production has increased more than 22-fold. However, given the amount of plastic consumed and the fact that not all of it can be recycled, plastic pollution is one of the fastest-growing threats in the world [10]. Polyethylene terephthalate (PET) is currently the most commonly used material for packaging (including bottles). The process of PET bottle recycling usually consists of several stages that generate different products: full quality recyclables, reduced quality recyclables, and waste [11]. After pre-cleaning, the compressed bottles are sent to grinders, where they are crushed into flakes with a target fraction of 4.0–12.0 mm, generating waste in the form of a PET powder (Figure 1a). After the grinding process, the PET flakes are washed again and the separation of fractions below 4.0 mm is also performed in this process. The PET flakes in the 4.0–12.0 mm fraction (Figure 1b) are most often recrystallized and, as a granulate, they are raw material for the production of packaging. The flakes below the fraction of 4.0 mm are not recrystallized for technical reasons, making them difficult to utilize (Figure 1c). According to data obtained from a leading recycling company in Poland, 4000 tons of PET bottles are recycled monthly, which results in 60 tons of by-product in the form of PET flakes with a fraction below 4.0 mm called “fines washed.” The creation of opportunities to manage such waste is an extremely important problem faced by recycling companies in Poland and around the world.

In the literature, there are various examples of the use of PET materials in concrete technology, but most often they concern PET fibers, i.e., intentionally prepared fibers (by the author or by the industry) rather than waste materials. The volume of fiber content with respect to fiber concrete is between 0.3% and 1.5%. Therefore, the procedure reuses a small amount of PET waste, and in addition, the waste must be deliberately prepared [12]. Evaluation of the application of these fibers (in these small proportions) indicates that the decrease in compressive strength of concrete with PET fibers is not significant (up to 10%), but the parameters related to tensile strength and, therefore, also flexural strength are significantly improved [13,14,15]. From a waste management point of view, it seems much more beneficial to use PET fractions as aggregate substitutes. The most significant conclusions that can be drawn from the available studies on the application of various types of PET are related to the influence of the amount of this substitute and its effect on the basic physical and mechanical properties, such as bulk density, compressive strength, or splitting tensile strength [16,17]; less frequently, the values of the modulus of elasticity or flexural toughness has been determined [12,18,19]. There is also a negligible number of studies in which non-destructive tests for this type of concrete are discussed [20]. The authors are rather consistent in their basic conclusions, which can be summarized as a statement that properties such as compressive strength, modulus of elasticity, or fresh mixture properties deteriorate with a percentage increase in PET waste application. However, for small amounts of waste, the decrease may not be significant, and some properties, such as splitting tensile strength, may be improved. Considering the growing interest in this type of composite, it becomes relevant to conduct simultaneous investigations of their properties using non-destructive techniques, which would allow for an ongoing assessment of the quality of larger batches of this material.

This study investigated concrete mixes that involved the addition of waste from the PET bottle recycling process, which cannot be recycled, or the spectrum of their application is limited. The design of modern building materials with a reduced environmental impact and consume fewer natural resources while disposing of non-recyclable waste is a necessary step in light of climate change and the European Union requirements being introduced. The implementation of waste into a new generation concrete mix, such as self-compacting concrete (SCC) mix, would reduce the environmental impact while maintaining fine mechanical properties [21].

## 2. Materials and Methods

### 2.1. Constituent Materials

#### 2.1.1. PET Recycled Aggregate

In this study, the PET fines washed material acquired from a leading PET bottle recycler in Poland was used. The physical and mechanical characteristics declared by the recycler are summarized in Table 1. Table 2 presents the maximum possible impurities of washed PET fines, although no significant impurities were observed in the material used for this study (only minor fragments of polyvinyl chloride (PVC) or polyethylene (PE)).

**Figure 2:**
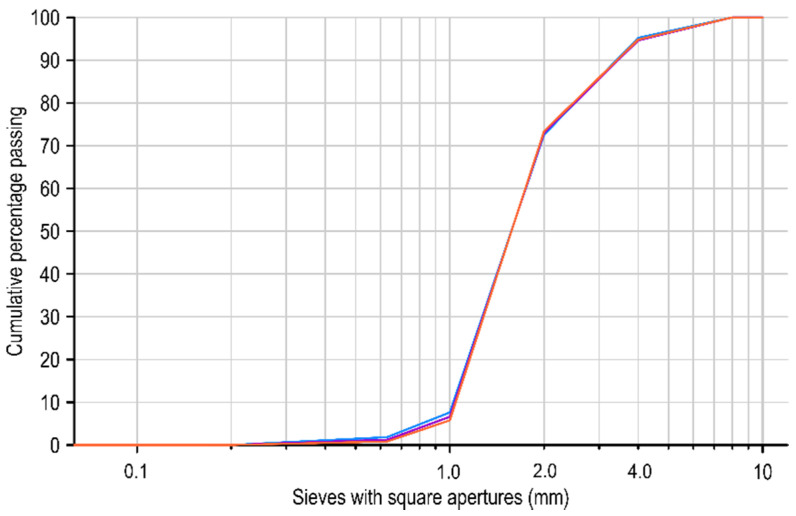
Grading curves for washed PET fines.

Given the relatively wide range of dimensions of the PET flakes declared by the manufacturer (0.5 to 4.0 mm), sieve analysis was performed according to standard [22] without the requirement of heating the aggregate at a temperature of 110 ± 5 °C to constant mass before further testing. The investigation was carried out for three samples taken from different parts of the collected test material, with each sample having a weight of 600 g. The results of the sieve analysis are provided in the form of grading curves for washed PET fines in Figure 2.

**Figure 3:**
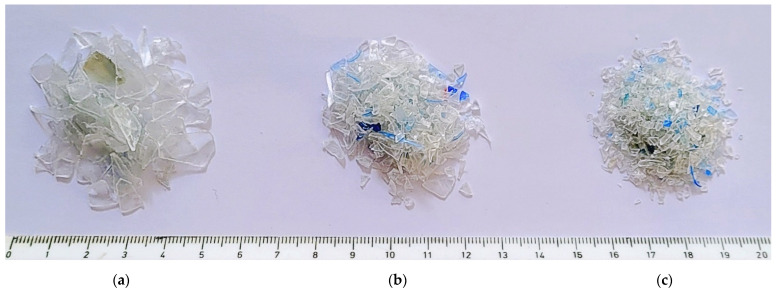
Washed PET fines sorted into fractions: (**a**) above 4.0 mm, (**b**) 2.0 to 4.0 mm, and (**c**) below 2.0 mm.

The grading curves of PET aggregate indicated that the particle size distribution for individual samples was nearly identical, and slight differences could be observed for the finest fractions. Fractions above 4.0 mm constituted approximately 5% of the material composition; fractions between 2.0 and 4.0 mm constitute 20% of the material; the largest fraction was between 1.0 and 2.0 mm, which represented almost 70% share; and the remaining 5% were fractions below 1.0 mm. The entire material was divided into three main fractions (above 4.0 mm, 2.0 to 4.0 mm, and below 2.0), as shown in Figure 3.

#### 2.1.2. Concrete Mixes

In this research program, 5 concrete mixes with different contents of PET flakes (fraction below 2.0 mm) were prepared, which was used as the fine aggregate. The SCC control mix was prepared with a replacement of 5%, 10%, 15%, and 20% of the mass of fine aggregate (sand). The mixtures were based on Portland composite cement CEM II/B-M of a strength category of 42.5 and normal strength gain (symbol N). The cement consisted of Portland clinker and the addition of fly ash and ground-granulated blast furnace slag in the amount of 21–35% according to EN 197 [23]. In the mixtures, two fractions of coarse aggregate (2–8 mm and 8–16 mm) and one fraction of fine aggregate (0–2 mm) were used. Gravel constituted the coarse aggregate, while sand and an appropriate amount of PET waste flakes were considered fine aggregate. Polycarboxylate with a polyethylene condensate defoamed-based admixture was selected as a superplasticizer.

The binder content was fixed at 440 kg/m^3^, which corresponded to a water-to-binder ratio of 0.36. The proportion of fine aggregate was fixed at 684 kg/m^3^ for all mixtures. The superplasticizer was dosed each time until sufficient flow parameters of the SCC were achieved. Detailed compositions of the mixtures are compiled in Table 3.

A total of 21 test elements (20 cubes with 150 mm sides and 1 cube with 100 mm sides) were prepared from each concrete mix, resulting in a total of 105 test elements.

### 2.2. Test Procedures

#### 2.2.1. Fresh Mixture Properties

All concrete mixtures were subjected to flow properties tests immediately after the mixing process. Flowability and plastic viscosity were evaluated using the slump flow test, during which, the time to 500 mm slump flow diameter (t_500_) and the diameter of the slump flow after completion of the flow were measured according to [24]. To evaluate passing ability, the L-box test with 3 bars was performed [25]. The segregation of the mixture was visually graded according to the standard [26].

#### 2.2.2. Compressive Strength

The compressive strength as the basic strength parameter of concrete was investigated according to [27] on cube samples with dimensions of 150 mm × 150 mm × 150 mm. The dimensions of the samples and the process of their preparation met the requirements of the standards [28,29]. The tests were performed after 28 days of concrete curing under laboratory conditions (temperature 20 ± 2 °C, humidity > 95%). Overall, 50 cubic samples were used to determine the compressive strength of the SCCs. Tests were performed using a universal testing machine (Walter + Bai AG, Löhningen, Switzerland).

#### 2.2.3. Splitting Tensile Strength

Another important strength parameter of concrete is the splitting tensile strength. Investigations of this aspect were performed according to standard [30] on cube specimens of dimensions 150 mm × 150 mm × 150 mm, as allowed by Annex A of this standard. Specially dedicated loading pieces and hardboard packing strips were used for the test. The splitting tensile strength was determined using Equation (1):

(1) $$f_{ct,split}=\frac{2F}{\pi Ld}\;,$$

where *F* is the maximum force, *L* is the contact line length of the specimen, and *d* is the claimed cross-sectional dimension. In total, 30 cubic samples were used to determine the splitting tensile strength of the SCCs. Tests were performed using a universal testing machine (Controls Group, Atlanta, GA, USA).

#### 2.2.4. Deformation Parameters

The determination of the deformation parameters was performed during the uniaxial compression test. These tests were performed using ISRM guidelines [31]. A hydraulic press with automatic piston feed, force registration, and longitudinal and transverse strain registration (Walter + Bai AG, Löhningen, Switzerland) was used to investigate the deformation parameters. Both the modulus of elasticity (*E*) and Poisson’s ratio (ν) were determined over the full range of linearity of the stress–strain characteristics (the so-called average modulus) of a given specimen. The modulus of elasticity was determined as the ratio of the difference in compressive stress difference ($\sigma_{2}-\sigma_{1}$) to the relative longitudinal strain ($\varepsilon_{2}-\varepsilon_{1}$) over a given measurement range using Equation (2):

(2) $$E=\frac{\sigma_{2}-\sigma_{1}}{\varepsilon_{2}-\varepsilon_{1}}\;,$$

Furthermore, Poisson’s ratio was determined as the ratio of transverse strain (*ε*_⊥_) to longitudinal strain ($\varepsilon_{∥}$) in a given compressive stress range using Equation (3):

(3) $$\nu =\frac{\varepsilon_{⊥}}{\varepsilon_{∥}}\;,$$

The deformation parameters were evaluated for three types of cylindrical core specimens: cores drilled in the direction of concrete casting and cores drilled in the direction perpendicular to concreting in the upper and lower parts of the cubic specimen. In general, 80 cylindrical samples were prepared to determine the deformation parameters of the SCCs.

#### 2.2.5. Ultrasound Test

Ultrasound examinations were performed using a PunditLab kit from the Proceq manufacturer (Schwerzenbach, Switzerland) with 54 kHz transducers with an automatic transmitter voltage. Prior to each use, the device was calibrated on a dedicated calibration rod with an ultrasound wave transit time of 25.4 μs. Measurements were performed with a coupling agent in the form of a chemically passive polyacrylate gel; both transducers’ heads were covered with a layer of gel before each measurement. The specimens were divided into two types: specimens for determining the mechanical properties of 150 mm × 150 mm × 150 mm cubes and specimens for determining the mechanical properties of 50 mm × 100 mm cylinders. For samples from both groups, the testing fields were prepared via gentle grinding. For samples in group 1, tests were made only for side surfaces in the central part of the samples, while for samples in group 2, four sections were defined: two along the direction of concrete casting and one in each of the upper and lower zones of the sample.

For each test section of a given sample, 3 measurements were performed and the average values of the ultrasound wave transit velocity (v) between the transducer heads were presented as the results. The tests were performed in relation to the standards EN 12504-4 [32] and EN 13791 [33].

#### 2.2.6. Sclerometric Tests

The sclerometric tests were conducted with a Proceq N-type Schmidt sclerometer (Switzerland). The Schmidt sclerometer allows for the surface hardness of the concrete to be measured from the indentation of an incident mass after its collision with the surface. In order to ensure proper stability of the tested samples and thus accuracy of the results, the samples were mounted in a vice. The tests were performed on the same surfaces as the ultrasound examinations. The testing fields were prepared via gentle grinding to achieve smoothness and flatness. For each test surface, nine measurements were performed and their results, properly arithmetically mediated, provided the sclerometric rebound index. The tests were performed in relation to the standards EN 12504-2 [34] and EN 13791 [33].

#### 2.2.7. X-ray Computed Tomography

X-ray computed tomography (X-ray CT) illustrating the distribution of PET flakes in each SCC was performed for selected samples. X-ray CT was performed using a GE (Boston, MA, USA) Phoenix v-tome-x m device.

The GE Phoenix v-tome-x m device together with VG Studio Max (version 2022.1, Heidelberg, Germany) software, enables the reconstruction and analysis of the internal structure of the element tested on the basis of a series of X-ray images obtained during a 360° rotation of the sample [35]. Table 4 describes the basic parameters of the test and the values used. Cuboid samples having dimensions of 50 mm × 50 mm × 100 mm, which were obtained by cutting 100 mm cube samples, were examined.

Beam hardening correction, automatic geometry calibration, and geometry optimization algorithms were applied during the reconstruction process. After the image reconstruction, separation of the PET flakes in a given sample from the 3D model was performed.

## 3. Results

### 3.1. Fresh Mixture Properties

The properties of the fresh SCC mixtures were determined using filling ability (slump flow) and passing ability (L-box) tests. Furthermore, the fresh visual segregation index was assessed. On the basis of the results, all mixtures were classified according to the European Guidelines for SCC [36]. As SCC with a 20% PET replacement did not meet the requirements of self-compacting concrete, but it was still flowable concrete, we refer to it as almost self-compacting concrete (ASCC). Moreover, as it was not self-compacting, the ASCC-P-20 mixture was carefully vibrated. Detailed test results are given in Table 5.

The main finding of the flow tests was that regardless of the investigated parameter, deterioration increased with the addition of PET flakes. The amount of superplasticizer was controlled to avoid segregation of the SCC mixture. Thus, despite the higher proportion of superplasticizer, the slump flow decreased with an increased proportion of PET flakes; simultaneously, the viscosity of the mixture (the time of slump flow up to 50 cm diameter) increased. The lower the natural aggregate content in the mix, the more difficult it was to maintain sufficient stability of the mix; therefore, bleeding was observed in all mixes with the addition of PET flakes. Consequently, due to the high probability of significant segregation, the mixture with 20% PET in relation to the fine aggregate mass did not reach the self-compacting guidelines. A reduction in the stability of the mixture was also observed during the L-box test. The L-box ratio deteriorated with the addition of PET flake aggregate as a result of the higher viscosity of the mixture. The authors of other studies on self-compacting mixes with PET aggregates drew analogous conclusions [37,38,39].

### 3.2. Destructive Tests of Hardened Concrete Properties

**Figure 4:**
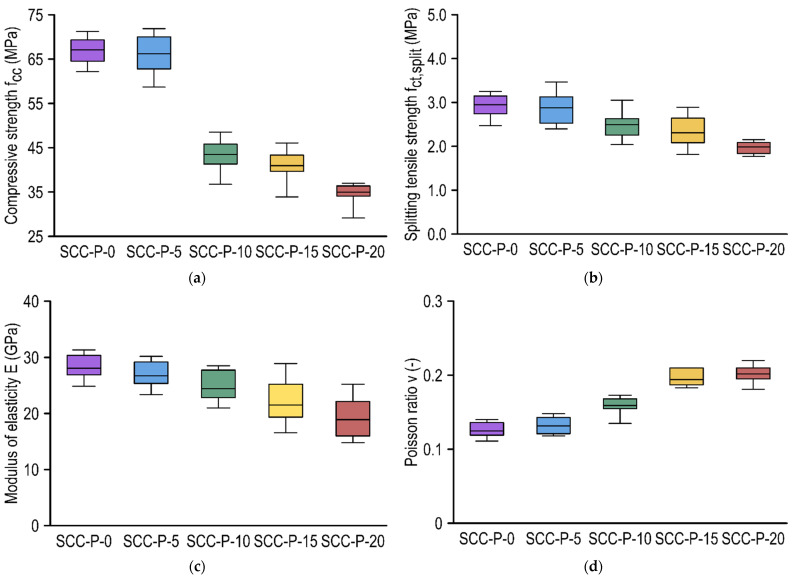
Results of hardened concrete parameters determined through destructive tests: (**a**) compressive strength, (**b**) splitting tensile strength, (**c**) modulus of elasticity, and (**d**) Poisson ratio.

The present research program involved the preparation of self-compacting concretes with recycled aggregate in the form of PET waste flakes. A series of tests were conducted on samples made from such concretes, including the determination of compressive strength, splitting tensile strength, modulus of elasticity, and Poisson ratio through the destructive test method. In addition, a series of non-destructive tests were performed, which are described in Section 3.3. The results of the destructive tests are presented graphically using box plots in Figure 4.

In whisker-box plots, the boxes are determined by quartiles (25% and 75%), the midpoint represents the mean of the results, and the minimum and maximum values of the test series are presented as whiskers. The results of the mechanical and deformation parameters are presented in relation to the type of mixture.

Compressive strength appeared to be significantly affected by the addition of PET waste flakes, starting with a 10% replacement of the fine aggregate (Figure 4a). The behavior of the control samples and those containing 5% PET aggregate may be considered equivalent, and the minor variations that appeared were a consequence of the scattering of the result. However, it should be noted that the scatter of compressive strength (COV) results for SCC-P-5 was higher than for SCC-P-0. This could have been associated with slight bleeding of a mixture. Sample analysis indicated that the results were symmetrically distributed relative to the average for concretes up to 10% PET replacement in the mix. For the SCC-P-15 and ASCC-P-20 concretes, the sample population was left-handed asymmetric, despite the overall lower values. Thus, the majority of the results were higher than the average, and the mean result decreased due to the high outliers (minimum value). The failure pattern of all samples after the compression test was consistent with the standard [27]; therefore, there was no reason to dismiss any results. Compared to the average compressive strength value for SCC-P-0, reductions of approximately 1.3%, 35.2%, 38.9%, and 47.8% occurred for concretes with 5%, 10%, 15%, and 20% PET replacements, respectively. The decrease in compressive strength may have been primarily related to the deterioration of the flow properties of SCC with recycled PET aggregate.

The splitting tensile strength decreased with the addition of PET aggregates to the SCC (Figure 4b). The decrease was similar in its course to the reduction in compressive strength but was not as significant. In terms of splitting tensile strength, SCC-P-0 and SCC-P-5 exhibited insignificantly different values that resulted from the scatter of the results. Some of the SCC-P-5 samples received higher splitting tensile strength values compared to the control samples; however, in general, its population was right asymmetric. Although the splitting tensile strength of SCC-P-15 was inferior to SCC-P-10, the distribution of the results in the populations suggested that these concretes performed similarly. The results for SCC-P-20 were significantly reduced compared to the control concrete; moreover, it is worth noting that the results did not show significant scatter and were symmetrically distributed around the mean. Compared to the mean value of the tensile strength splitting of SCC-P-0, reductions of 2.4%, 15.6%, 21.7%, and 32.5% occurred for concretes with PET replacements of 5%, 10%, 15%, and 20%, respectively.

Young’s modulus determined in compression decreased with the addition of PET aggregate (Figure 4c). Due to the adopted test method, the results deteriorated compared to those available when testing according to the EN procedure [40]. The reduction in the modulus of elasticity of SCC-P-5 compared to the control concrete was more significant than for the parameters mentioned above and reached almost 5%. It is also worth noting that the test results for all concretes except SCC-P-20 were left-handed asymmetric; therefore, most of the results were above the mean. The largest scatter of results was obtained for concretes with the highest PET aggregate content (15% and 20%). Compared to the average modulus of elasticity value of SCC-P-0, reductions of 5.0%, 13.2%, 23.5%, and 32.7% on average were observed for concretes with PET replacements of 5%, 10%, 15%, and 20%, respectively.

The higher the PET percentage in the mixture, the higher the Poisson ratio in compression (Figure 4d). Its change corresponded to the other parameters determined. Compared to the average Poisson ratio of SCC-P-0, reductions of 5.4%, 27.4%, 55.5%, and 61.7% were found for concretes with 5%, 10%, 15%, and 20% PET replacements, respectively.

In the literature, there is some research on destructive tests of self-compacting concretes with PET aggregate in terms of compressive strength, splitting tensile strength, and modulus of elasticity [37,41,42,43]. Nevertheless, the issue has been significantly better recognized in ordinary concretes or research has been conducted on concretes with the addition of PET fibers. In general, the trends shown in this study coincided with the literature; a broader comparison is presented in Section 4.

### 3.3. Non-Destructive Tests of Hardened Concrete Properties

**Figure 5:**
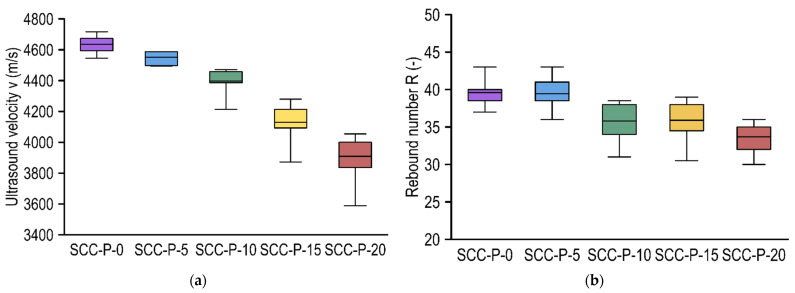
Non-destructive test results: (**a**) ultrasound velocity v and (**b**) rebound number R.

Non-destructive test series were performed on the samples, including the determination of the rebound number and ultrasound wave transition velocity. As in the case of destructive tests, the results are presented graphically using box plots (Figure 5). The test results are presented in relation to the type of mixture.

For both sclerometric and ultrasound tests, a decrease in the test result value was observed with increasing PET aggregate content. For ultrasound tests, the relationship of the decrease in wave transition velocity with the increase in PET flake content was more linear than for the sclerometric tests. One can also observe that the decrease in the ultrasound wave velocity was accompanied by an increase in the coefficient of variation, which was primarily caused by registering single results significantly lower than the average (especially for SCC-P-10, SCC-P-15, and SCC-P-20). The reduction in the velocity of the ultrasound wave transition for successive concretes was well correlated with the decrease in density as the proportion of PET replacement in the concretes increased. Compared to the average ultrasound wave transition velocity of SCC-P-0, reductions of 2.0%, 5.2%, 10.0%, and 15.8% on average occurred for concretes with 5%, 10%, 15%, and 20% PET replacements, respectively. In the case of the bulk density of hardened concrete, a density of 2360.3 kg/m^3^ was recorded for SCC-P-0 and, on average, reductions of 2.4%, 3.8%, 5.7%, and 9.5% occurred for concretes with 5%, 10%, 15%, and 20% PET replacements, respectively.

In the sclerometric tests, the variations were not linear; for SCC specimens without and with 5% PET replacement, the rebound numbers differed minimally and the scatter was slightly less for the control specimens. For SCC-P-10 and SCC-P-15, a significant decrease was reported relative to the control samples, and the values were also close to each other. Significantly lower results were observed, which may have been due to the location of the PET flakes close to the sample surface, which may have directly affected the decrease in surface hardness, and the highest coefficient of variation was observed here. The lowest rebound number results were recorded for SCC-P-20 specimens. Compared to the average rebound number of SCC-P-0, decreases of 0.4%, 9.6%, 9.4%, and 14.9% on average were observed for concretes with 5%, 10%, 15%, and 20% PET replacements, respectively. Similar correlations were obtained for compressive strength; hence, the conclusion is that the cause may have also been due to a significant deterioration in the flow properties of the fresh mixture.

In the literature, there are individual works on non-destructive testing of self-compacting concretes [37,43], although they refer only to ultrasound tests. The issues related to non-destructive tests are also the subject of the design code for structural concretes, allowing for the development of correlation relationships between destructive and non-destructive tests, which are presented and discussed in Section 4.

### 3.4. X-ray CT Scans

**Figure 6:**
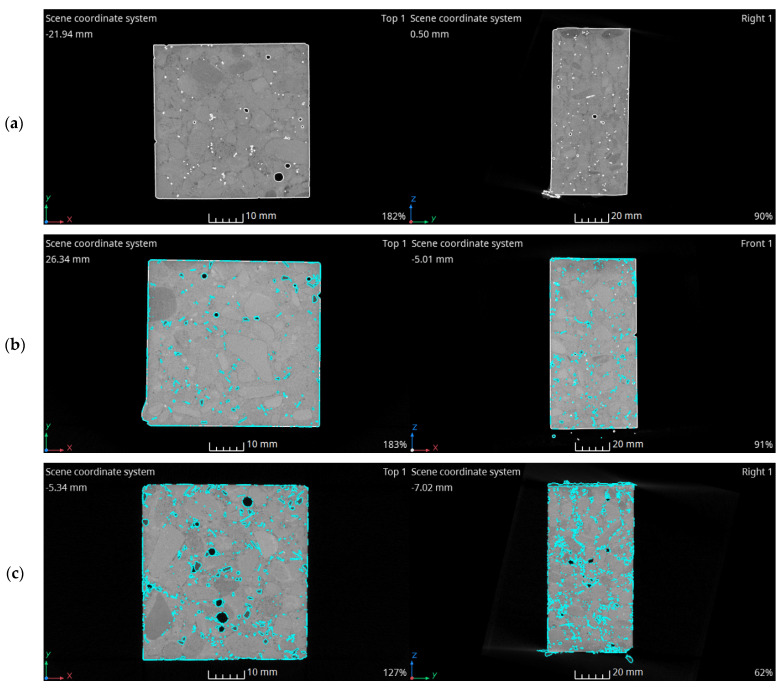

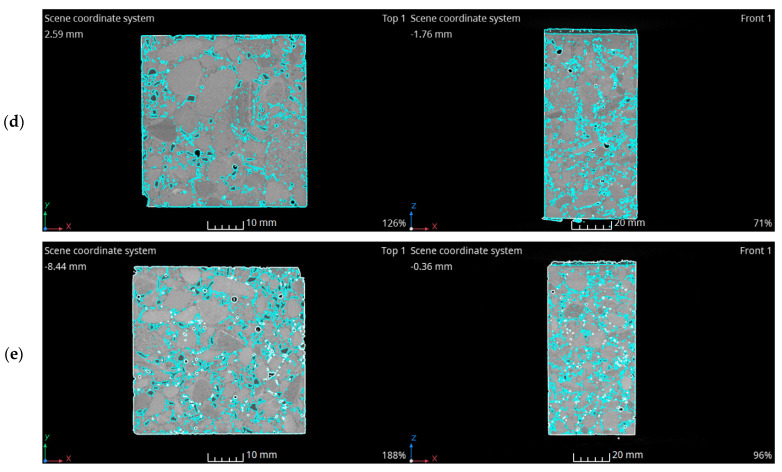
X-ray CT scans—identification of PET flakes in a concrete sample: (**a**) SCC-P-0, (**b**) SCC-P-5, (**c**) SCC-P-10, (**d**) SCC-P-15, and (**e**) SCC-P-20.

X-ray CT scans provided a view of the distribution of PET flakes in the concrete specimens. To visualize them more clearly in the figures, an analysis was performed to isolate the voxels in a given grey value range corresponding to the PET material. Figure 6 presents transverse and longitudinal sections through the tomographic images in which white was used to distinguish air pores in the material and cyan was used to distinguish PET flakes. The cyan around certain pores, e.g., in the SCC-P-10 image, was related to the grey value of the cement matrix around the air bubbles. Analysis of the image allowed us to conclude that PET flakes were evenly distributed throughout the sample volume and did not clump together, which was connected with the proper stability of the mixture. In PET concretes, a reduced density material layer was observed in the top part of the samples. This phenomenon was evidence of concrete mixture bleeding, which was also observed during the determination of the fresh visual stability index. The layer increased proportionally to the observed bleeding during the mix examination (up to approximately 2 mm in the tomographic images). PET flakes also appeared in the reduced density layer; however, no increased flake distribution was observed in the higher part of the samples. Another phenomenon worth noting is that air pores tended to be trapped underneath horizontal flakes.

## 4. Discussion

### 4.1. A Discussion of the Results with the Literature Findings

**Figure 7:**
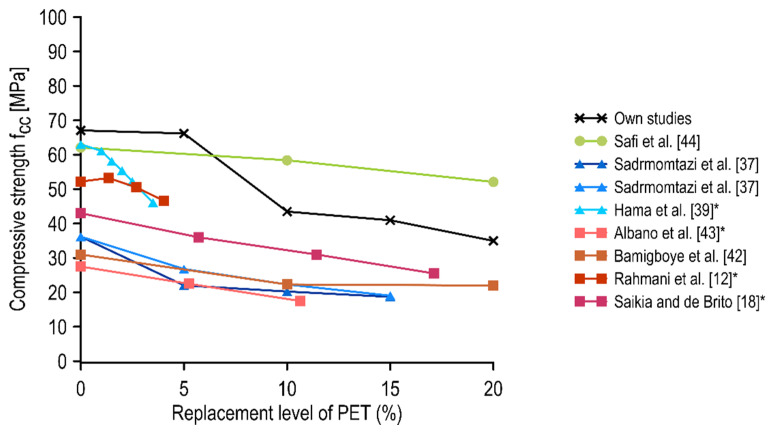
Variations of 28-day compressive strength of different concretes with respect to the replacement level of PET. * Converted from volume replacement.

In concrete materials technology, compressive strength is taken as the basic mechanical characteristic. In the present study, it was found that the modification of the fine aggregate to up to 5% of the PET aggregate had no significant effect on the compressive strength of the SCC. A considerable reduction in compressive strength was observed for mixtures containing at least 10% PET flakes relative to the mass of sand, while for subsequent percentages, the effect was not as pronounced. These results were compared with data from the available literature and presented in a summary graph in Figure 7. The following types of concretes were analyzed: traditional concrete with PET flakes (red tone lines) [12,18,42,43], self-compacting mixtures with PET flakes (blue tone lines) [37,39], and self-compacting mixture with PET granules (green line) [44]. The results of the present study are given in black. In the case of the literature studies that assumed volumetric substitution of fine aggregate, the proportion of substitution was converted to mass replacement relative to sand mass in order to properly compare the results of the study.

The mixture containing PET granules did not show a significant decrease in compressive strength up to 10% PET granule content. This may have been related to the favorable shape of this PET aggregate, as well as the generally higher compressive strength of concrete (high strength concretes are generally characterized by better quality).

In general, SCC mixes with PET flakes exhibited a reduction in compressive strength, even in the case of a small replacement percentage. It should be noted that the control concretes varied in compressive strength between experiments; therefore, the comparison was made on the variations rather than the values themselves. Self-compacting concrete appeared to be more prone to a decrease in compressive strength as the proportion of PET aggregate increased. This was related to the deterioration of flow properties and the increased sensitivity of self-compacting mixtures to segregation when adequate technological rigor is not maintained. In the case of the highest replacement percentage of fine aggregate in Hama et al. [39], which was an approximately 5% mass replacement, there was a 27% decrease in compressive strength compared to the control concrete. Sadrmomatzi et al. [37] observed a reduction of approximately 40% at a sand mass replacement of 10%. In the present study, for concrete with a 10% replacement of fine aggregate with PET aggregate, a decrease of 35% was observed. The greater reduction in compressive strength found in [37] compared to the findings of our study could have been the result of the larger PET fraction used in the mixtures (up to 4.75 mm), which corresponded to the replacement of coarse aggregate. In the analyzed experiments, a further increase in PET content did not affect the reduction of compressive strength as significantly.

The compressive strength of traditional concretes also tended to decrease as the proportion of PET in the mix increased. For lower-strength normal concretes, these reductions were more significant, although not as pronounced for self-compacting lower-strength concretes. In the case of the study by Rahmani et al. [12], the total reduction achieved was up to 11% (comparing concrete with approximately 5% replacement by sand mass to the control one). However, it is worth noting that for a minor sand mass substitution (approximately 1.5%), the compressive strength of concrete increased. In the cases of the other researchers analyzing normal concrete with PET aggregate, a contribution of 10% by weight of sand resulted in an average reduction in compressive strength of 31%, which was slightly lower than the result obtained in our studies.

**Figure 8:**
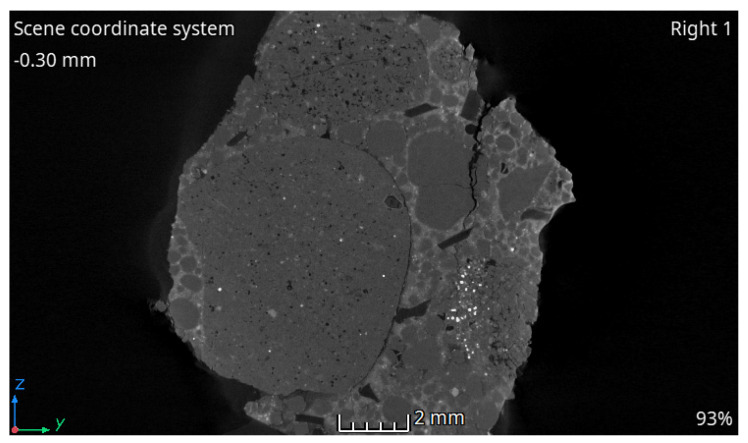
X-ray CT scan—section of a fragment of an SCC-P-15 sample after compression.

A noteworthy hypothesis is that the decrease in strength of concrete with PET recycled aggregate is related to the increased softness of the material compared to natural aggregate, which was proposed by Faraj et al. [17]. The review authors stated that during loading, PET aggregate behaves as air voids inside the cement matrix, resulting in the initiation of cracks around the particles. This claim was verified using a tomographic image of the SCC-P-15 fragment subsequent to compressive strength tests, which illustrated the propagating cracks surrounding the particles (Figure 8).

Furthermore, on the basis of SEM studies, it was concluded by other authors that as the proportion of PET aggregate increases, the porosity of concrete and the width of the poor quality interfacial transition zone (ITZ) increases. Kangavar et al. [19] reported that the structure of concrete incorporated with PET granules appeared to be more cavernous with larger air bubbles when the substituted volume exceeded 30%. In comparison to other types of plastic, PET aggregate generates the smallest ITZ with lower quality due to its surface roughness resulting from the processing of this material [45]. Based on the literature, the lower compressive strength of concretes with higher proportions of PET is related to the lower bond between the cement matrix and PET and the increased porosity of concrete, which is also reflected in the lower density of concrete.

**Figure 9:**
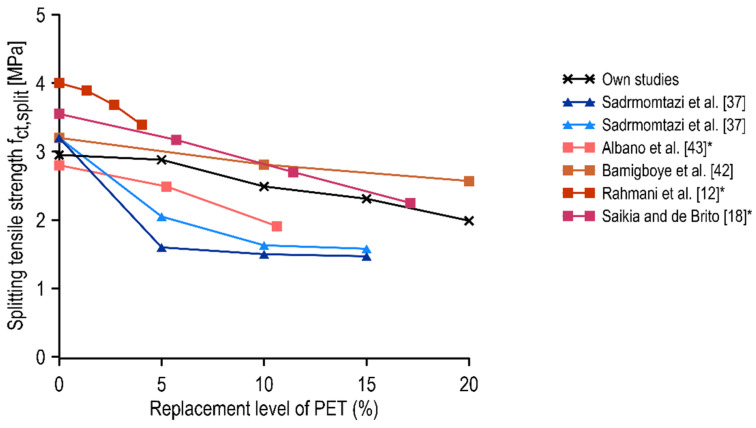
Variation of 28-day splitting tensile strength of different concrete with respect to the replacement level of PET. * Converted from volume replacement.

The second characteristic that is of great importance for concrete composites is tensile strength. In the present study, analogous to compressive strength, it was found that substitution of fine aggregate to PET aggregate up to 5% did not significantly affect this property. The subsequent decrease in the splitting tensile strength with increasing PET content can be considered to be relatively linear. These results were compared with available literature data and presented in a summary graph in Figure 9, where the amount of data to be compared was less than for compressive strength due to the significantly different research approaches of other authors. Comparisons were made between traditional concrete mixes with PET flakes (red tone lines) [12,18,42,43] and self-compacting mixes with PET flakes (blue tone lines) [37]. The results of the present study are given in black. In the case of the studies that assumed volumetric substitution of fine aggregate, the proportion of substitution was converted to mass replacement relative to sand mass in order to properly compare the results of the study.

All traditional concrete mixes with PET aggregate exhibited a similar linear decrease in splitting tensile strength with an increased proportion of PET aggregate. In the case of the replacement of 5% fine aggregate with PET aggregate, both Albano et al. [43] and Saikia et al. [18] obtained a decrease in splitting tensile strength of about 11%, while Rahmani et al. [12] reported a greater decrease of 17% compared to the control concrete. In [37], for SCC, the highest decrease in splitting tensile strength (for a 5% replacement of sand with PET aggregate) was recorded, up to 36% for samples with fly ash content and 50% in the case of samples containing only cement as a binder. In all the analyzed studies, the further decrease in splitting tensile strength for higher PET aggregate content was not as significant. It should be noted that in the case of concretes with PET aggregate, the increase in splitting tensile strength that is visible in the application of concrete with PET fibers, e.g., in [13], is not usually observed. This is a direct consequence of the different, more spherical, and irregular shapes of the PET aggregate used.

The ultrasound results obtained were similar to those reported in the literature. Albano et al. [43] tested normal concretes with PET aggregate and found wave transition velocities of 3000 m/s to 4200 m/s, which was lower than in this study. However, they also reported a decrease in wave transition velocity with increasing PET flake content and the magnitude of these variations did not change with concrete aging (tests conducted after 7 to 60 days of curing). For self-compacting concrete, Sadrmomtazi et al. [37] found that the control SCC had a wave transition velocity of 4700 m/s, while for concretes containing a different proportion of PET additives, they were 3830 m/s to 4580 m/s; these findings are very similar to those obtained herein.

The empty spaces created by the air pores and PET particles attenuate the ultrasound wave due to the acoustic impedance. When the ultrasound wave passes through different media (materials), it is partially reflected and transmitted; therefore, its velocity decreases. First of all, the decrease in the ultrasound wave velocity is caused by the reduction in concrete density [46]. Moreover, as noted by Albano et al. [43], due to the fact that the ultrasonic wave velocity is a function of the elastic properties and volume concentrations of the constituents when replacing natural fine aggregate with PET, the ultrasound wave velocity must decrease.

### 4.2. Comparison of NDT and DT Testing

**Figure 10:**
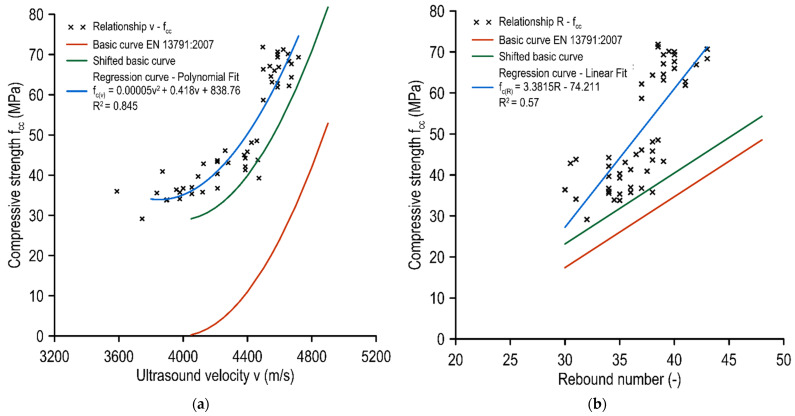
A comparison of non-destructive tests results to the compressive strength with normative basic curves: (**a**) ultrasound vs. compressive strength test results and (**b**) sclerometric vs. compressive strength test results.

The tests performed on the mixtures with different PET replacements indicated significantly varying results of ultrasonic wave transit velocity tests. Therefore, it was possible to correlate the compressive strength with the velocity of the ultrasound wave and relate these results to the standard basic curve presented in Figure 10a. The correlation of these results with a polynomial function can be described as strong (R^2^ = 0.845). The basic curve, on the other hand, seemed to be strongly underestimated relative to the results obtained. However, it should be considered that the basic curve presented in EN 13791 [33] is the lower envelope and should, in principle, be placed below the results obtained from destructive tests. Furthermore, a standard procedure for the correction of the basic curve was performed, which involved the change in the basic curve by Δ*f* (Equation (4)), which consists of the average difference obtained between the destructive tests results of the samples and the results obtained from the basic curve $\delta f_{m\left(n\right)}$ reduced by the product of the standard deviation s and a factor *k*_1_ that depends on the number of pairs of results (in this research, *k*_1_ = 1.48):

(4) $$\Delta f=\delta f_{m\left(n\right)}-k_{1}\cdot s,$$

The shifted basic curve provides a better representation of the relationships described. There is only a single point located under the curve (thus the estimation using this function would be highly secure). The analyses carried out indicated that it was necessary to adopt a limitation of the curve application (both shifted and correlation) similarly to the basic curve. For the shifted basic curve, it was 3900 m/s to 4800 m/s, and for the acquired correlation curve, it was 3700 m/s to 4600 m/s. Crucially, the correlation function exhibited a pattern very similar to that of the basic and shifted functions in terms of direction.

For sclerometric tests, analogous considerations were made with respect to the standard [9]. First, a correlation analysis of the NDT test results with DT was performed. A moderate-strength linear correlation was obtained (R^2^ = 0.57). As in the case of ultrasound examinations, these results were summarized in a combined graph with a basic curve and a shifted basic curve (Figure 10b). It can be observed that the basic and shifted basic curves had a decreased slope compared to the curve derived from the linear regression obtained. For minor values of the rebound number, the difference in compressive strength gained from the standard basic curves compared to the correlation curve was not significant and it grew as the rebound number increased. The basic curve was found to be below all the measurements obtained and for the shifted basic curve, as for ultrasound, there was only a single result below the curve. Therefore, it might be applicable with a high level of safety (with the remark that the higher the rebound number, the more underestimated the result). It should be emphasized that sclerometric tests are conducted on the surface and do not comprehensively describe the strength properties of the tested materials. However, their simplicity and promptness favor the application of this method for regular in situ concrete quality control.

### 4.3. Implementation of PET Aggregate in New Generation Concretes

The use of PET aggregate in the form of flakes, which is a by-product of the recycling process (it should be noted that the fraction used in this study is not recyclable into granulate, which constitutes a valuable raw material), is far more beneficial from an environmental point of view than the use of granulates or specially prepared fibers. One can assume that the acquisition of PET flakes in fractions above 4.0 mm (used in the production of PET granulate) is inevitably connected with the production of waste in the form of smaller-fraction flakes and PET powder. Therefore, the costs associated with PET bottle treatment need to be borne anyway, which improves the economic profile of SCCs with PET aggregates compared to SCCs with PET fibers. However, as demonstrated in numerous studies, the use of PET fibers in small quantities (less than 2% by volume) has the potential to improve the mechanical properties of concrete, especially in terms of compressive strength and flexural strength, including SCCs [47,48]. Nevertheless, it should be emphasized that due to the hydrophobic nature of PET, the flow properties of SCC mixtures deteriorate in the case of both PET fibers addition and sand replacement with PET aggregate. Therefore, caution is required when implementing larger amounts of plastic into self-compacting mixes in order to ensure that segregation phenomena do not occur.

Given that the world’s annual production of concrete is steadily increasing and currently reaches approximately 32 billion tons per year [1], and its production requires non-renewable natural resources, the exploration of substitutes, even if partial, is highly valuable. On average, about 300 kg of sand is used to produce one ton of concrete; hence, the annual consumption of this raw material is about 9.6 billion tons. Considering the replacement of 5% of natural sand with waste PET aggregate of the fraction below 2.0 mm, material savings of 480 million tons per year of this resource could be reached.

Nowadays, SCCs are considered to be a special type of concrete used in structures with dense reinforcement and complex geometries. However, given the further aspects of environmental and social assessment, it seems inevitable that the utilization of mixtures that do not require additional technological processes (such as vibration and troweling) will increase in demand. It is essential to investigate the incorporation of waste materials into new generation concretes in order to effectively implement these solutions in the future.

## 5. Conclusions

This paper discusses the possibility of using PET waste flakes of the fraction below 2.0 mm in self-compacting concrete mixtures. The presented results and their juxtaposition with data from the literature provide the following conclusions:

- PET aggregates in self-compacting concrete were uniformly distributed regardless of their content in the mix.
- The higher the PET content in the mix, the lower the density of the concrete, and therefore, the velocity of the ultrasonic wave and the rebound number of the sclerometer. The non-destructive test results acquired relatively good correlations with the compressive strength test results.
- The replacement of fine aggregate with PET recycled aggregate negatively affected the parameters of both fresh mixes and hardened concretes. In the case of the concrete with the highest aggregate replacement, the decreases compared to the control concrete were 9.5%, 47.8%, 32.5%, 32.7%, and 61.7% in terms of bulk density, compressive strength, splitting tensile strength, modulus of elasticity, and Poisson ratio, respectively. The ASCC-P-20 also did not meet the requirements of self-compaction.
- However, the replacement of 5% of natural aggregate with PET aggregate of the appropriately selected fraction was possible for SCC and did not cause significant variations in the physical and mechanical properties: 2.4%, 1.3%, 2.4%, 5.0%, and 5.4% in terms of bulk density, compressive strength, splitting tensile strength, modulus of elasticity, and Poisson ratio, respectively.

Research on the properties of SCC mixtures with PET aggregates in the form of flakes should be further developed to determine properties such as corrosion resistance, frost resistance, water penetration resistance, the durability of concrete or bond to reinforcing steel, and the bond to subsequent layers of concrete. In addition to the differences in the obtained basic research results relative to the literature data, as important steps forward in this research, one can indicate the determination of correlation curves for non-destructive testing and the determination of the Poisson’s ratio, which is not commonly referred to, but can be useful in the future structural design using SCC with PET (especially for FEM calculations).

## Figures and Tables

**Table 1:** Physical and mechanical characteristics of washed PET fines.

Physical and Mechanical Characteristics	Value
Specific density	~1.35 g/cm^3^
Bulk density	~550 kg/m^3^
Flakes size	0.5 to 4.0 mm
Intrinsic viscosity	0.62 to 0.75 dL/g
Melting point	App. 250 °C
Color	Clear, light blue, blue, green

**Table 2:** Impurities PET fines washed.

Impurities	Value
Metal	<1%
Paper/labels	<10%
Polyolefine (PE)	<5%
PVC	<0.2%
Share in dust	<5%
Glue	Yes
Caustic soda	Yes

**Table 3:** The composition of the mixes used per cubic meter.

Ingredients	Unit	SCC-P-0	SCC-P-5	SCC-P-10	SCC-P-15	SCC-P-20
CEM II/B-M 42.5 N	kg	440	440	440	440	440
Water		160	160	160	160	160
Sand (0–2 mm)		684	650	615	581	547
PET waste flakes (0–2 mm)		-	34	69	103	137
Gravel (2–8 mm)		510	510	510	510	510
Gravel (8–16 mm)		560	560	560	560	560
Superplasticizer		7.54	7.00	7.00	5.75	4.00
PET aggregate level	-	0%	5%	10%	15%	20%

**Table 4:** Parameters of the X-ray CT test.

Parameter	Value
Source voltage	160 kV
Source current	180 μA
Filter	0.2 mm copper (Cu) filter
Exposure time	250 ms
Number of X-ray pictures used to reconstruct a 3D model	3100

**Table 5:** Properties of the fresh SCC mixtures.

Fresh Mixtures Properties	SCC-P-0	SCC-P-5	SCC-P-10	SCC-P-15	ASCC-P-20
Slump flow (mm)	725	710	660	600	510
Slump flow class—SF	SF 2	SF 2	SF 2	SF 1	-
Slump flow time T_50_ (s)	2.5	4.5	5.5	7.0	3.0
Viscosity class—VS	VS 2	VS 2	VS 2	VS 2	VS 2
L-box ratio	0.88	0.87	0.86	0.81	0.62
L-box class	PL 2	PL 2	PL 2	PL2	-
Fresh visual stability index	0	1	1	1	2

## Data Availability

The data that support the findings of this study are available from the corresponding author upon request.
